# A Review of Diterpenes from Marine-Derived Fungi: 2009–2021

**DOI:** 10.3390/molecules27238303

**Published:** 2022-11-28

**Authors:** Peng Qiu, Jinmei Xia, Haitao Zhang, Donghai Lin, Zongze Shao

**Affiliations:** 1 Marine Biomedical Research Institution, Guangdong Medical University, Zhanjiang 524023, China; 2 Key Laboratory of Marine Biogenetic Resources, Third Institute of Oceanography, Ministry of Natural Resources, Xiamen 361005, China; 3 Guangdong Key Laboratory for Research and Development of Natural Drugs, Guangdong Medical University, Zhanjiang 524023, China; 4 Key Laboratory for Chemical Biology of Fujian Province, MOE Key Laboratory of Spectrochemical Analysis & Instrumentation, College of Chemistry and Chemical Engineering, Xiamen University, Xiamen 361005, China

**Keywords:** diterpene, marine, fungi, bioactivity

## Abstract

Marine-derived fungi are important sources of novel compounds and pharmacologically active metabolites. As an important class of natural products, diterpenes show various biological activities, such as antiviral, antibacterial, anti-inflammatory, antimalarial, and cytotoxic activities. Developments of equipment for the deep-sea sample collection allow discoveries of more marine-derived fungi with increasing diversity, and much progress has been made in the identification of diterpenes with novel structures and bioactivities from marine fungi in the past decade. The present review article summarized the chemical structures, producing organisms and biological activities of 237 diterpenes which were isolated from various marine-derived fungi over the period from 2009 to 2021. This review is beneficial for the exploration of marine-derived fungi as promising sources of bioactive diterpenes.

## 1. Introduction

**Figure 1:**
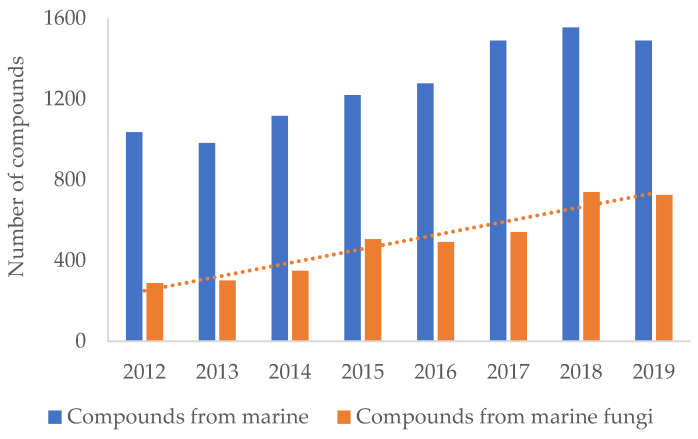
Numbers of new compounds from marine and marine-derived fungi from 2012 to 2019, data adapted from the serial review articles of Blunt et al. ([1,2,3,4,5,6,7,8]).

As the largest part of the Earth’s surface area, the ocean contains resources worthy of in-depth exploration. Marine-derived fungi, which are rich sources of secondary metabolites, have great potential for the discovery of bioactive compounds. The number of new compounds derived from marine fungi is increasing every year, from 287 in 2012 [1] to 724 in 2019 [2]. The contribution of marine fungi in marine-derived compounds is also increasing, from 27.7% in 2012 to 48.6% in 2019 [3,4,5,6,7,8] (Figure 1).

The marine fungi-derived compounds are of very diverse types, which include alkaloids [9], terpenes [10], polyketones [11], peptides [12], etc. As a major class of secondary metabolites of marine fungi, terpenes show many excellent activities [13]. Diterpenes are a group of terpenes with various bioactivities and rich structural diversity [10].

There are quite a few review articles on the isolation, structure elucidation, and biological activities of diterpenes. Hanson James R. has been publishing reviews of new diterpenoids discovered every year since 1984 [14] and the latest report was published in 2009 [15]. After that, Hanson began to limit the scope of the reviews to terrestrial diterpenoids and the first report was published in 2011 [16]. His serial review articles were then published almost every year and the latest one was published in 2019 [17]. These review articles offer important information for newly found diterpenoids of terrestrial origin. In contrast, no such systematic and up-to-date review articles are available for marine-derived diterpenes.

In recent years, more and more research articles have reported works on the discovery of new diterpenes from marine-derived fungi. Expectedly, a review of these works will help to better understand recent discoveries and advances in this field. Herein, we summarize the structures and activities of newly discovered diterpenes derived from marine fungi in the past 13 years from 2009 to 2021.

## 2. Characteristics of Diterpenes from Marine-Derived Fungi

**Figure 2:**
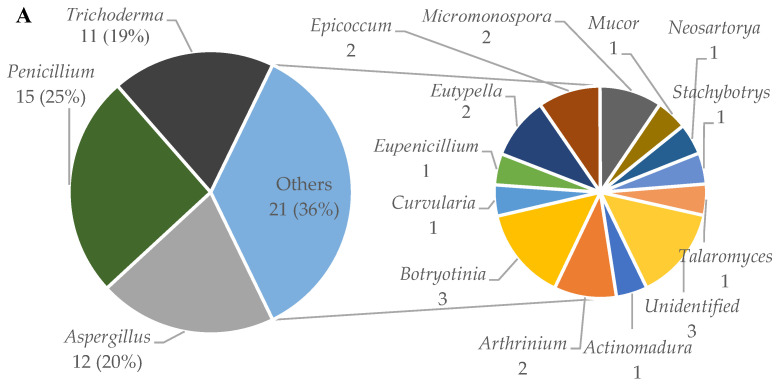

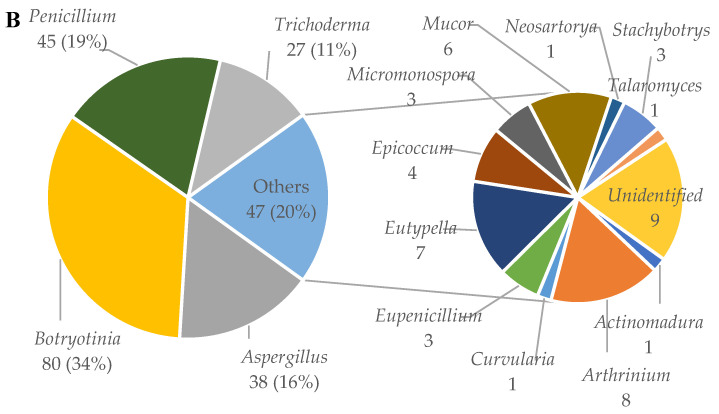
Distribution of the number of papers reporting marine fungi-derived diterpenes (**A**) and the number of diterpenes (**B**) by fungal genus.

From 2009 to 2021, 237 new diterpenes were isolated from 47 strains of marine fungi that belong to 15 genera (*Actinomadura*, *Arthrinium*, *Aspergillus*, *Botryotinia*, *Curvularia*, *Eupenicillium*, *Eutypella*, *Epicoccum*, *Micromonospora*, *Mucor*, *Neosartorya*, *Penicillium*, *Stachybotrys*, *Talaromyces*, and *Trichoderma*). The pie chart in Figure 2A shows the distribution of the genera of the fungi covered in the 59 articles that reported newly discovered diterpenes. In these articles, *Penicillium* (25%), *Aspergillus* (20%), and *Trichoderma* (19%) are the most frequently studied genus. A total of 38 articles reported diterpenes from fungi of these three genera. Regarding the number of compounds, *Botryotinia* (34%), *Penicillium* (19%), and *Aspergillus* (16%) are the most productive, producing 80, 45, and 38 of new diterpenes, respectively (Figure 2B).

**Figure 3:**
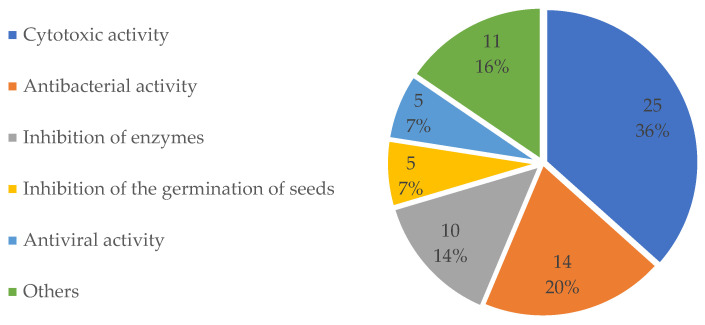
Percentages of different kinds of activities compared to the whole occurrence of activities of bioactive diterpenes derived from marine fungi.

Of the 237 new compounds, 68 compounds were reported to possess various bioactivities (Table 1). A total of 70 pieces of activity data are available since both compounds **38** and **202** were reported to possess two kinds of bioactivities. Cytotoxic activity is the most reported bioactivity, with 25 of 70 compounds (compounds with more than one kind of activity were also counted more than once) (36%, Figure 3) being active. Antibacterial activity (20%), inhibition of enzymes (14%), antiviral activity (7%), and inhibition of the germination of seeds (7%) were the next, with the number of active compounds being 14, 10, 5, and 5, respectively.

## 3. Isolation, Structures, and Bioactivities of Marine Fungi-Derived Diterpenes

### 3.1. Actinomadura

**Figure 4:**
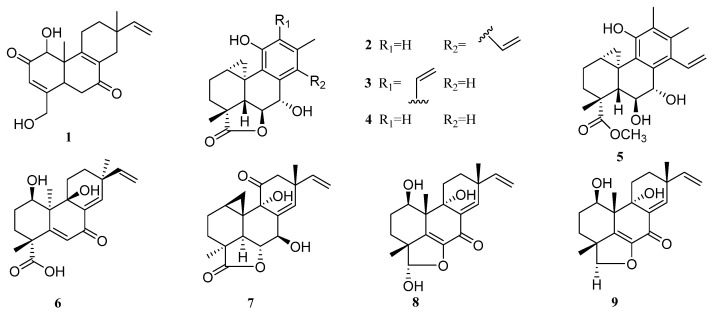
Chemical structures of diterpenes (**1** from *Actinomadura* sp., **2**–**9** from *Arthrinium* sp.).

Only one new diterpene was reported to be produced by the genus *Actinomadura* since 2009 (**1**, Figure 4). Compound JBIR-65 (**1**) was obtained from the sponge-derived fungus *Actinomadura* sp. SpB081030SC-15 [49]. This is the first report of a diterpene isolated from the genus *Actinomadura*. This work found that compound JBIR-65 possessed an ability to protect neuronal hybridoma N18-RE-105 cells from L-glutamate toxicity with an EC_50_ value of 31 µM (Table 1).

### 3.2. Arthrinium

Eight new diterpenes were reported for the genus *Arthrinium* (**2**–**9**, Figure 4). Five diterpenoids, arthritis A–D (**2**–**5**) and myrocin D (**6**) were isolated from the sponge-derived fungus *Arthrinium* sp. [50]. The bioactivity test revealed that myrocin D (**6**) had antitumor activity, it inhibited vascular endothelial growth factor A (VEGF-A)-dependent endothelial cell sprouting with an IC_50_ value of 2.6 µM, while the IC_50_ value for the positive control, sunitinib, was 0.12 µM.

In addition, in another fungus, *Arthrinium sacchari*, which was isolated from the sponge surface, researchers obtained three new diterpenes: myrocin D (**7**), libertellenone E (**8**), and libertellenone F (**9**) [55]. Antitumoral potentials of compounds **7**–**9** were tested in an in vitro angiogenesis assay against human umbilical vascular endothelial cell (HUVEC) sprouting induced by VEGF-A, but no positive result was obtained.

It is worth noticing that both compounds **6** and **7** were named myrocin D. They were identified from different strains of *Arthrinium* by different researchers. The coincidence may be explained by the close timing of submission and acceptance of the two articles, which were published in different journals [50,55].

### 3.3. Aspergillus

**Figure 5:**
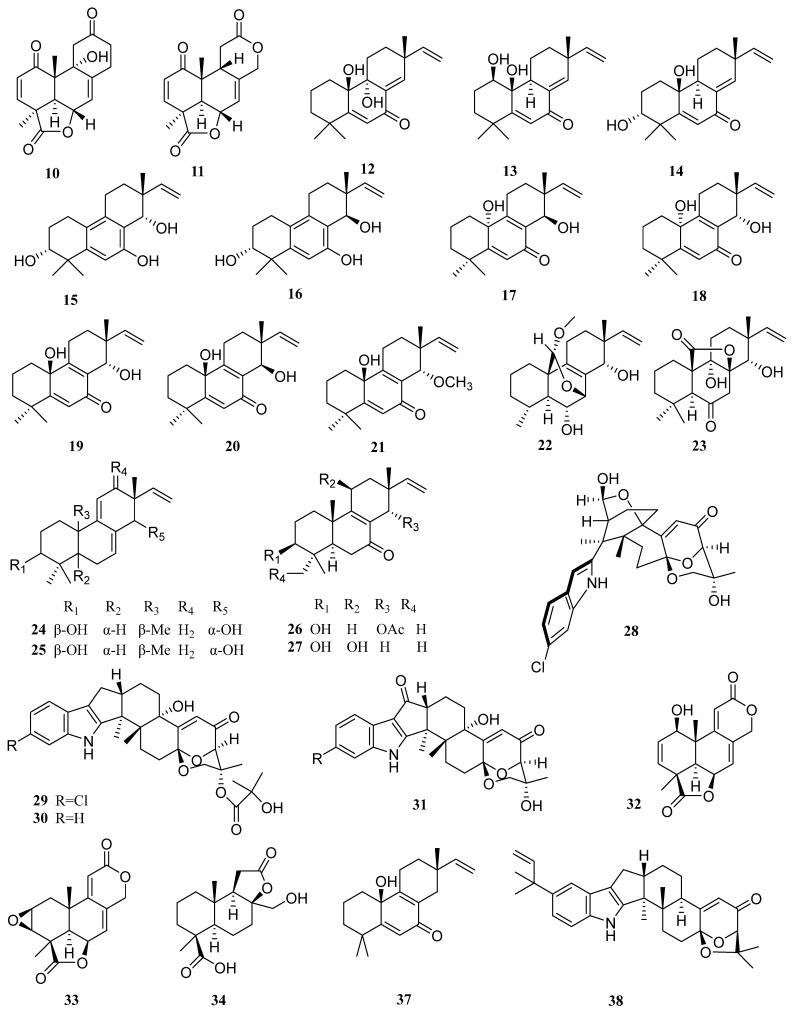

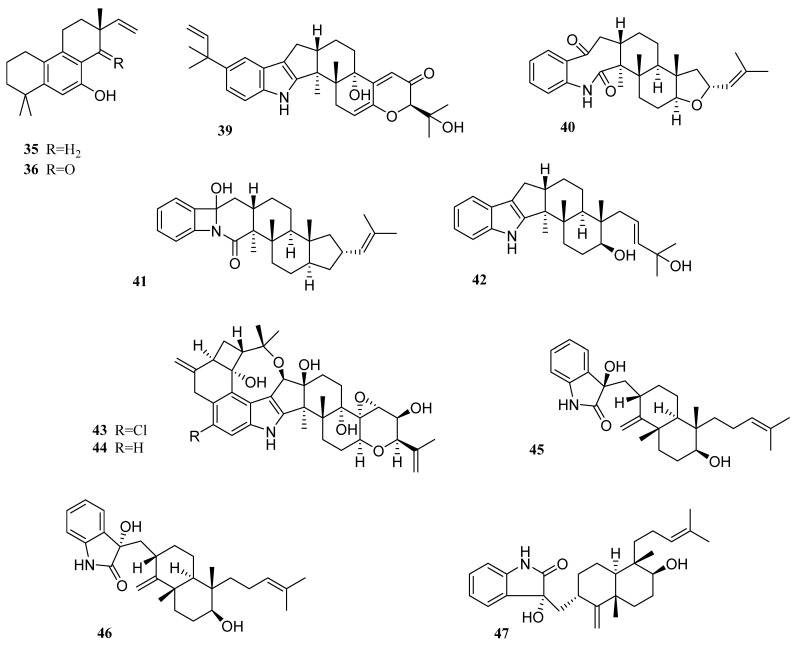
Chemical structures of diterpenes (**10**–**47** from *Aspergillus* sp.).

From 2009 to 2021, 12 articles reported the discovery of 38 new diterpenes (**10**–**47**, Figure 5) from marine *Aspergillus*, accounting for over one-fifth of the total articles. *Aspergillus* of marine origin is an important source of active compounds. More than 170 of 232 compounds isolated from marine *Aspergillus* from 2006 to 2016 showed cytotoxic and antimicrobial activities [56].

Li et al. isolated the fungus *Aspergillus wentii* SD-310 from deep-sea sediments. Further investigation of its products led to the isolation of 18 new diterpenes (**10**–**27**), including two new tetranorlabdane diterpenes, asperolides D (**10**) and E (**11**) [33]. Compound **10** had moderate inhibitory activities against *Edwardsiella tarda*, with an MIC value of 16 µg/mL (Table 1). Chloramphenicol and ampicillin were used as positive controls, with MIC values being 8.0 and 2.0 µg/mL, respectively.

The 18 new compounds also include nine 20-nor-isopimarane diterpenoids, aspewentins D−L (**12**–**20**), and a new methylated derivative, aspewentin M (**21**) [34,42]. The pimarane diterpenes were reported to have a wide range of biological activities including antimicrobial, antifungal, antiviral, phytotoxic, phytoalexin, cytotoxic, and antispasmodic effects [57]. An activity test showed that compound **12** and compounds **14**–**16** have inhibitory activities against aquatic pathogens *Edwardsiella tarda*, *Micrococcus luteus*, *Pseudomonas aeruginosa*, *Vibrio harveyi*, and *V. parahemolyticus*, each with an MIC value of 4.0 µg/mL. Compounds **12** and **16** showed inhibitory activities against the plant pathogen *Fusarium graminearum*, with MIC values of 2.0 and 4.0 µg/mL, respectively. Notably, these two compounds were more potent than the positive control, amphotericin B (MIC of 8.0 µg/mL). Compounds **17** and **18** showed inhibitory effects against zoonotic pathogenic bacteria between human and aquatic animals such as *Escherichia coli*, *Edwardsiella tarda*, *Vibrio harveyi*, and *V. parahaemolyticus*. Compound **21**, which may prove useful as an antifungal agent, exhibited potent antimicrobial activities against some plant pathogenic fungi, such as *Fusarium graminearum*.

Another 6 of the 18 new compounds produced by the strain *Aspergillus wentii* SD-310 were isopimarane diterpenoids, wentinoids A–F (**22**–**27**) [57]. This is the first report on the isolation of isopimarane diterpenoids from the species *Aspergillus wentii*. The antibacterial activities of these compounds were evaluated. Compound **22** had inhibitory effects on *Fusarium. oxysporum* f. sp. *lycopersici*, *Phytophthora parasitica*, *Fusarium graminearum*, and *Botryosphaeria dothidea*, with MIC values of 4.0, 8.0, 1.0, and 4.0 µg/mL, respectively, which were comparable to that of the positive control, amphotericin B (MIC values being 1.0, 2.0, 1.0, and 2.0 µg/mL, respectively) [57].

Indole diterpenoids are an important group of diterpenoids with diverse bioactivities [58]. The mining of the metabolites of the sponge-derived fungus *Aspergillus candidus* HDN15-152 resulted in the isolation and identification of four new indole diterpenoids, ascandinines A–D (**28**–**31**) [18]. Compounds **29**–**31** are diterpenes with rare 6/5/5/6/6/6/6 fused ring systems. Compound **30** displayed an anti-influenza virus A (H1N1) activity with an IC_50_ value of 26 µM (ribavirin as the positive control, IC_50_ = 31 µM), while compound **31** showed cytotoxicity against HL-60 cells with an IC_50_ value of 7.8 µM (Table 1) [18].

Sun et al. obtained three new norditerpenoids, asperolides A–C (**32**–**34**), from a brown algal-derived fungus *Aspergillus wentii* EN-48 [59].

New diterpenes were also found from the fermentation of the fungus *Aspergillus wentii* na-3, which was isolated from the surface of *Sargassum alagl* [51]. A chemical epigenetic manipulation strategy was used to turn on the silent metabolic pathways. A histone deacetylase (HDAC) inhibitor, suberoylanilide hydroxamic acid (SAHA), was added to the medium, and three new norditerpenes (**35**–**37**) were obtained [51].

In addition, the inhibitory effects of compounds **35**–**36** on the growth of one marine zooplankton (*Artemia salina*) and three marine phytoplankton species (*Chattonella marina*, *Heterosigma akashiwo*, and *Alexandrium* sp.) were evaluated. The results showed that compound **36** inhibited the growth of *Artemia salina* with an LC_50_ of 6.36 µM, and compound **35** was active against *Chattonella marina* and *Heterosigma akashiwo*, with LC_50_ values of 0.81 and 2.88 µM, respectively.

Sun et al. isolated two indole diterpenes, (2*R*, 4b*R*, 6a*S*, 12b*S*, 12c*S*, 14a*S*)-4b-Deoxy-β-aflatrem (**38**), and (2*R*, 4b*S*), 6a*S*, 12b*S*, 12c*R*)-9-Isopentenylpaxilline D (**39**), from the *Penaeus vannamei*-derived fungus *Aspergillus flavus* OUCMDZ-2205 [19]. Compounds **38** and **39** were cytotoxic to the A-549 cell cycle in the S phase with IC_50_ values of 10 µM. Besides, compound **38** had an inhibitory effect on the kinase PKC-*β* with an IC_50_ value of 15.6 µM, which was effective in attenuating vascular complications of diabetes.

Two new indole diterpenes derivatives asporyzins A–B (**40**–**41**), one new indole diterpenes asporyzin C (**42**), and three known related indole diterpenes were isolated from the red algae-derived fungus *Aspergillus oryzae.* These three new compounds did not show antibacterial activity against *Escherichia coli* and antifungal activity against plant pathogens *Colletotrichum lagenarium* and *Fusarium oxysporum* [60].

Zhang et al. isolated two indole diterpenes, 19-hydroxypenitrem A (**43**) and 19-hydroxypenitrem E (**44**), from a red alga-derived fungus *Aspergillus nidulans* EN-330 [61]. In the assay for antibacterial activity against pathogens *Edwardsiella tarda, Vibrio anguillarum*, *Escherichia coli*, and *Staphylococcus aureus*, compound **43** showed activities with MIC values of 16, 32, 16, and 16 µg/mL, respectively, comparing with 16, 0.5, 2, and 2 µg/mL of the positive control, chloramphenicol [35]. Compound **44** lacks a chlorine atom compared with compound **43** and is much less active, indicating that the Cl substitution on C-6 might enhance the antimicrobial activity and the cytotoxic activity against brine shrimp (*Artemia salina* L.) larvae, which was consistent with previous studies [61].

Marwa Elsbaey et al. isolated two new oxoindolo diterpenes, anthcolorin G (**45**) and anthcolorin H (**46**), from a mangrove-derived fungus *Aspergillus versicolor* [20]. Their biological activities were evaluated on HeLa cells, and only compound **46** showed activity with an IC_50_ value of 43.7 µM.

Zhang et al. isolated a new indole diterpene, (3*R*, 9*S*, 12*R*, 13*S*, 17*S*, 18*S*)-2-carbonyl3-hydroxylemeniveol (**47**) from a marine fungus *Aspergillus versicolor* ZZ761 [36]. The biological activity test using *Escherichia coli* and *Candida albicans* showed MIC values of 20.6 and 22.8 µM, respectively.

### 3.4. Botryotinia

**Figure 6:**
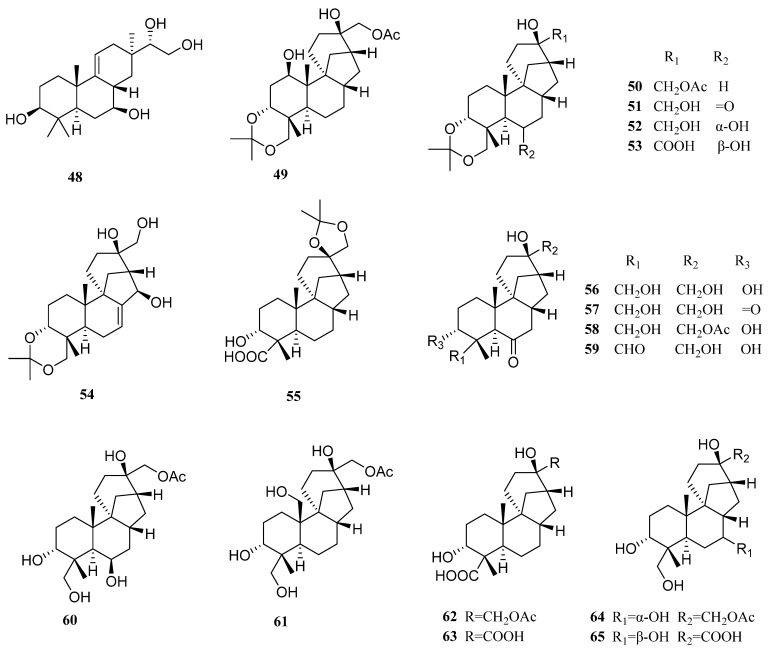

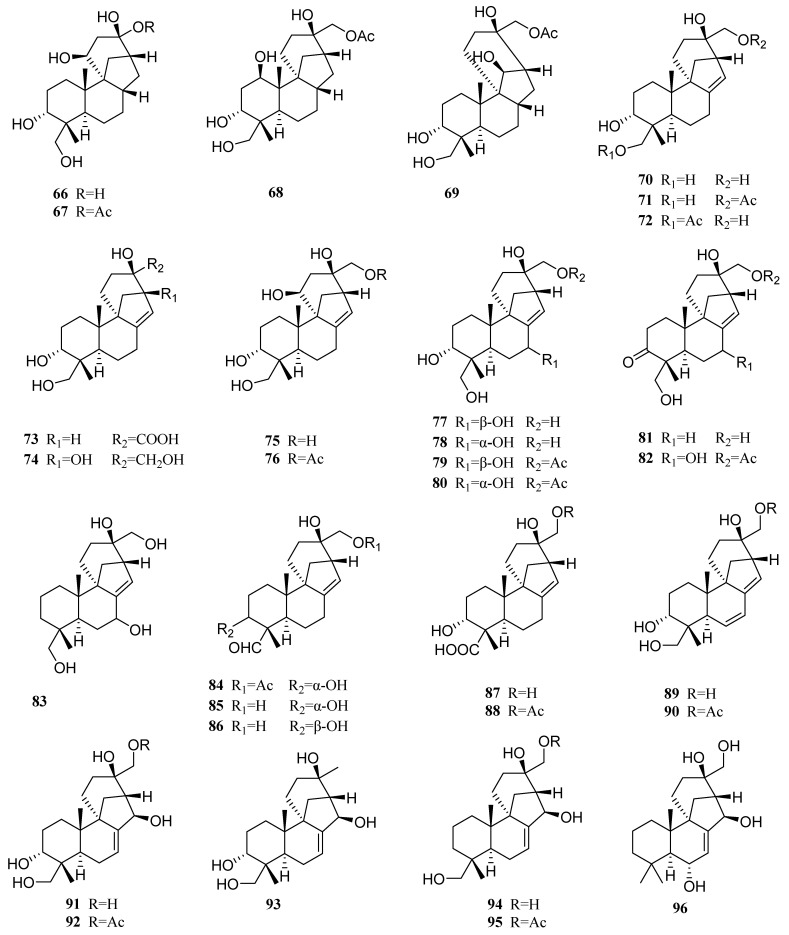

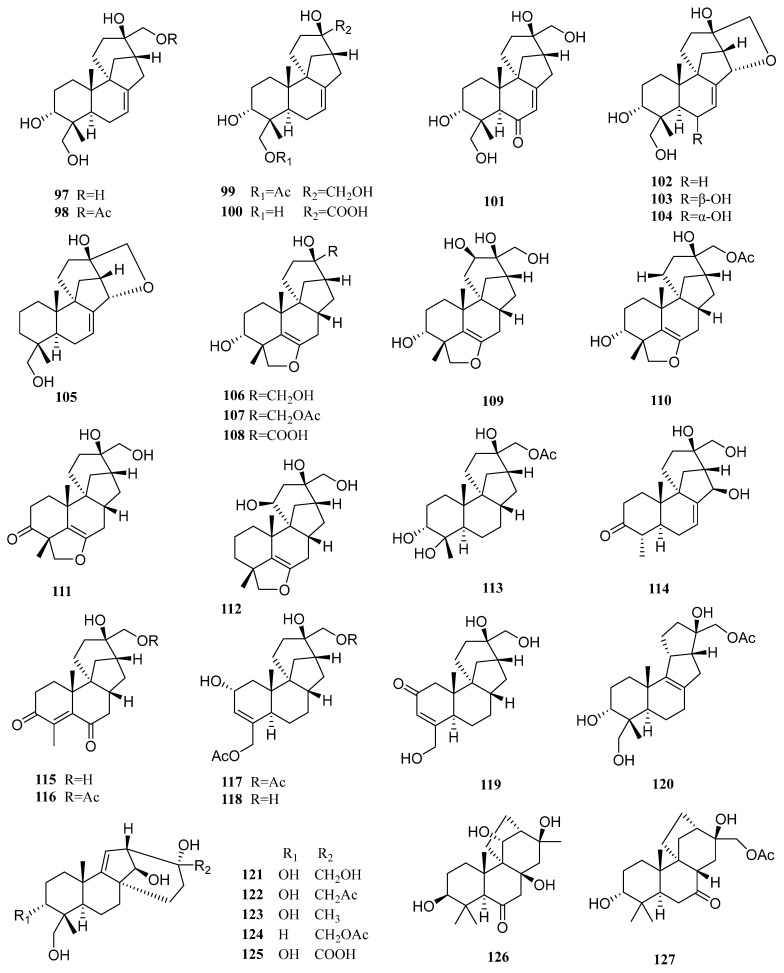
Chemical structures of diterpenes (**48**–**127** from *Botryotinia* sp.).

*Botryotinia* has previously been studied more as a plant pathogenic fungus than as a natural product producer [62]. The investigation of a marine *Botryotinia* strain, *Botryotinia fuckeliana* MCCC 3A00494, led to the isolation of 80 new diterpenes (**48**–**127**, Figure 6).

*Botryotinia fuckeliana* MCCC 3A00494 was isolated from the deep sea at −5572 m. A new pimarane diterpenoid with a Δ^9(11)^ double bond, botryopimarene A (**48**), which was rarely discovered in the pimarane family, was obtained from its fermentation [63].

Another 71 new diterpenes A1–A71 (**49**–**119**), all belonging to aphidicolin congeners, were also obtained from the same strain [21]. Compounds **102**–**106** and **107**–**113** are novel 6/6/5/6/5 pentacyclic aphidicolanes featuring tetrahydrofuran and dihydrofuran rings, respectively. In addition, compounds **114**–**119** are rare noraphidicolins. Significantly, aphidicolin A8 (**56**) showed good activities against T24 and HL-60 cells, with IC_50_ values of 2.5 and 6.1 µM, respectively. Thus, compound **56** can serve as a potent cytotoxic lead compound.

The potential of the strain MCCC 3A00494 in producing diverse diterpenes was more than that. Further investigation of its products led to the isolation of eight more new diterpenes. They represent three new carbon skeletons with 6/6/5/5 (**120**), 6/6/5/6 (**121**–**125**), and 6/6/6/5 (**126**–**127**) tetracyclic scaffolds. In terms of biological activity, compound **120** showed anti-allergic activity with an IC_50_ value of 0.2 mM (loratadine as the positive control with an IC_50_ of 0.1 mM) [53].

### 3.5. Curvularia

**Figure 7:**
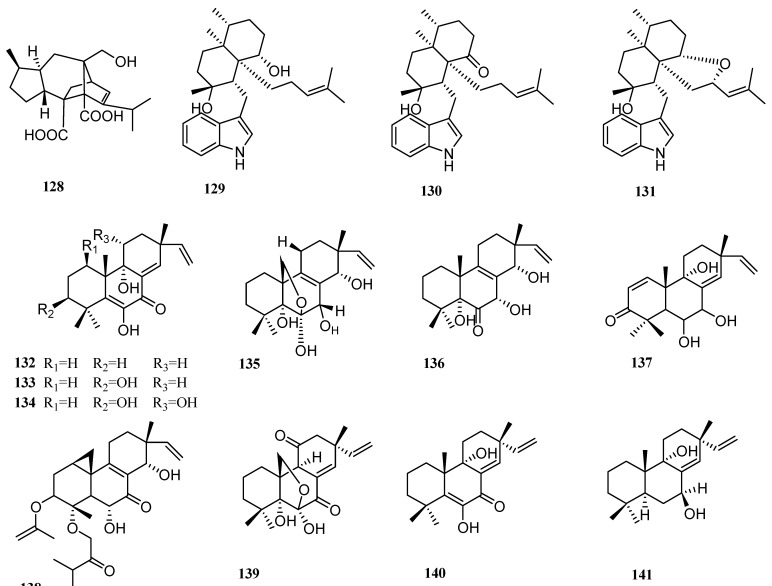

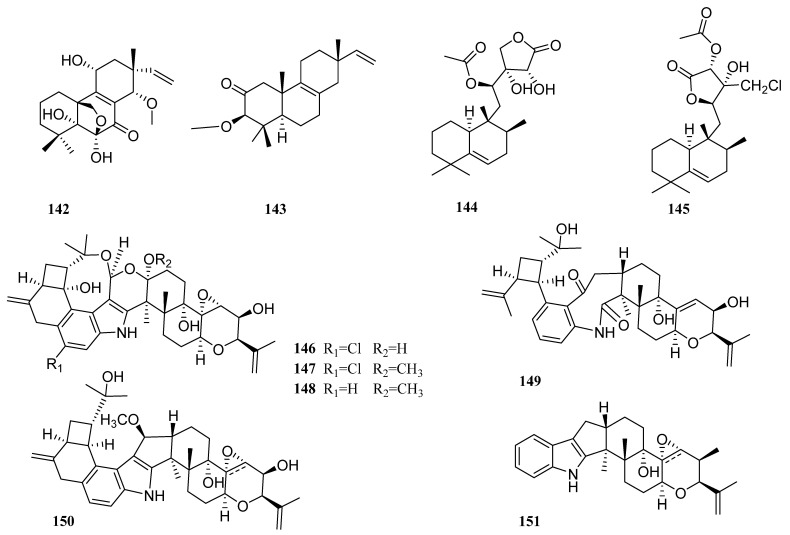
Chemical structures of diterpenes (**128** from *Curvularia* sp., **129**–**131** from *Eupenicillium* sp., **132**–**138** from *Eutypella* sp., **139**–**142** from *Epicoccum* sp.,**143**–**145** from *Micromonospora* sp., and **146**–**151** from *Mucor* sp.).

Only one new diterpene was produced by the genus *Curvulari* since 2009 (**128**, Figure 7). An investigation of extracts from the coral-derived fungus *Curvularia hawaiiensis* TA26-15 afforded one new sordaricin tetracyclic diterpene, sordaricin B (**128**), together with two known analogs, moriniafungin and sordaricin [64]. Their antifungal, antibacterial, and antiviral activities were tested. Moriniafungin and sordaricin showed antifungal activities against *Candida albicans* ATCC10231 with MIC values of 24 and 18 µM, whereas sordaricin B (**128**) did not show observable biological activity.

### 3.6. Eupenicillium

Zheng et al. isolated three new indole diterpenes, penicilindoles A–C (**129**–**131**, Figure 7), from a mangrove-derived fungus *Eupenicillium* sp. HJ002 [22]. Cytotoxic activities of all compounds against human A-549, HeLa, and HepG2 cell lines were evaluated by the MTT method. Compound **129** displayed biological activities against human A-549 and HepG2 cell lines with IC_50_ values of 5.5 and 1.5 µM, respectively. These values for the positive control, adriamycin, were 0.002 and 0.1 µM, whereas for 5-fluoracil, 36.8 and 76.9 µM, respectively.

### 3.7. Eutypella

Seven diterpene compounds were produced by the genus *Eutypella* since 2009 (**132**–**138**, Figure 7). Sun et al. isolated five new oxygenated pimarane diterpenes from a marine sediment-derived fungus *Eutypella scoparia* FS26, which were named scopararanes C–G (**132**–**136**). The biological activities of these compounds were evaluated on three human cell lines, including SF-268 (human glioma cell line), MCF-7 (human breast adenocarcinoma cell line), and NCI-H460 (human non-small cell lung cancer cell line) [65]. The results showed that compounds **132** and **133** exhibited weak cytotoxicity against the MCF-7 cell line with IC_50_ values of 35.9 and 25.6 µM, respectively.

Liu et al. isolated two new pimarane-type diterpenes, named scopararanes H–I (**137**–**138**), from a marine sediment-derived fungus *Eutypella* sp. FS46, which was collected at a depth of −292 m [24]. Compound **138** showed moderate inhibitory activities against NCI-H460 and SF-268 cell lines with IC_50_ values of 13.59 and 25.31 µg/mL, respectively.

### 3.8. Epicoccum

The genus *Epicoccum* generated four diterpenes (**139**–**142**, Figure 7). Xia et al. isolated three new pimarane-type diterpenes, compounds **139**–**141**, from a marine-derived fungus *Epicoccum* sp. HS-1. All isolated compounds were tested for cytotoxicity against KB (human epidermis carcinoma cell line) and KBv200 (a classic multidrug-resistant cell line) cells [25]. Compounds **139** and **140** inhibited the growth of KB cells with IC_50_ values of 3.51 and 20.74 µg/mL, and the growth of KBv200 cells with IC_50_ values of 2.34 and 14.47 µg/mL, respectively. As the positive control, cisplatin showed cytotoxic activities against KB and KBv200 cell lines with IC_50_ values of 0.96 and 0.76 µM, respectively.

Xia et al. also isolated another new isopimarane diterpene from the same strain, naming it isopimarane diterpene (**142**) [43]. In the bioactivity assay, compound **142** exhibited α-glucosidase inhibitory activity with an IC_50_ value of 4.6 µM. Isopimarane diterpenes were reported to have biological activities such as antiviral, cytotoxic, etc. This is the first report on the α-glucosidase inhibition activity of isopimarane diterpenes. Compound **142** might be applied for the treatment of type 2 diabetes.

### 3.9. Micromonospora

The genus *Micromonospora* produced three new diterpene compounds (**143**–**145**, Figure 7). Mullowney et al. isolated a novel Δ^8,9^-pimarane diterpene, named isopimara-2-one-3-ol-8,15-diene (**143**), from a sediment-derived fungus *Micromonospora* sp. [66].

*Micromonospora* sp. WMMC-218 is a fungus derived from the marine ascidian *Symlegma brakenhielmi*. LC-MS-based metabolomics was used and showed that the secondary metabolite profile of the strain is unique. Further investigation of the fermentation led to the isolation of two new halimane-type diterpenoid micromonohalimanes A (**144**) and B (**145**) [37]. This is the first time that halimane-type diterpenes isolated from the genus *Micromonospora*. In terms of activity, compound **145** displayed an inhibitory effect on the methicillin-resistant *Staphylococcus aureus* with an MIC value of 40 µg/mL, compared with the MIC value of 1 µg/mL for the positive control, vancomycin.

### 3.10. Mucor irregularis

The genus *Mucor* yielded six diterpenes (**146**–**151**, Figure 7), which are all indole diterpenes. They were discovered by Gao et al. and were named rhizovarins A–F (**146**–**151**). The producing strain *Mucor irregularis* QEN-189 was isolated from mangroves. Among these compounds, rhizovarins A–C, with the unique 4/6/6/8/5/6/6/6/6 nine-ring structure and a rare acetal, have the most complex structure among the indole diterpenes reported before 2016 [26].

Activities of compounds **146**–**151** were assessed on human A-549 and HL-60 cancer cell lines. Compounds **146**, **147**, and **151** showed biological activities against the A-549 cancer cell line with IC_50_ values of 11.5, 6.3, and 9.2 µM, respectively, compared with 0.30 µM of adriamycin as the positive control. Compounds **146** and **147** were active against the HL-60 cancer cell line with IC_50_ values of 9.6 and 5.0 µM, respectively, compared with 0.067 µM of adriamycin.

### 3.11. Neosartorya

**Figure 8:**
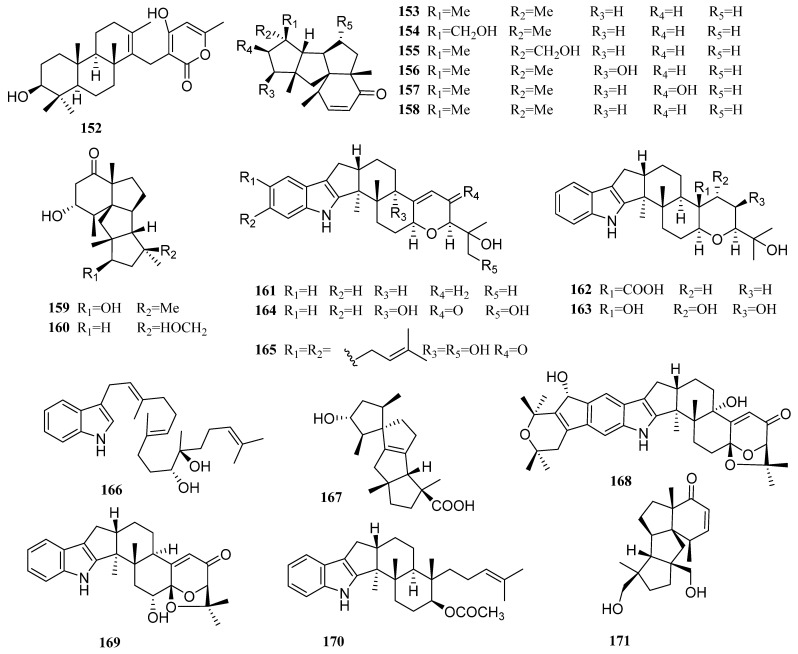

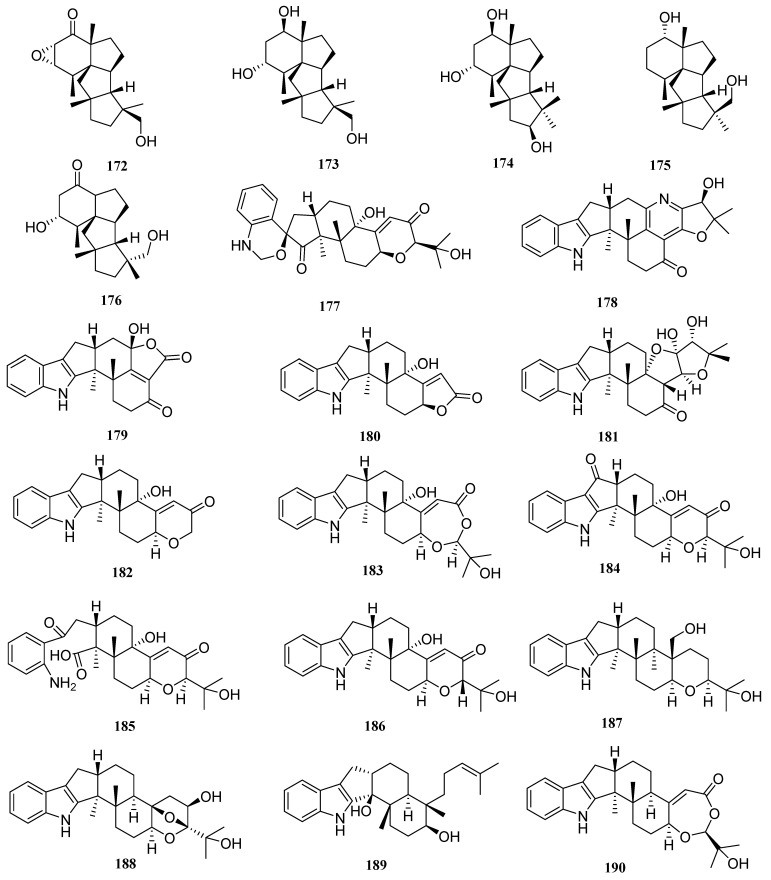

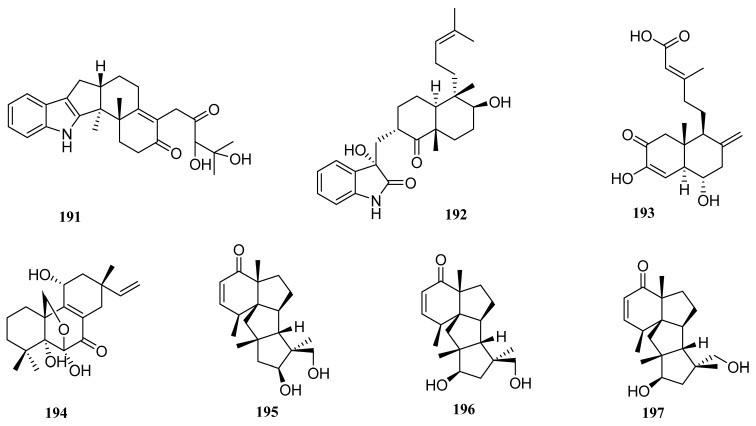
Chemical structures of diterpenes (**152** from *Neosartorya* sp., **153**–**197** from *Penicillium* sp.).

Only one diterpene was produced by the genus *Neosartorya* since 2009 (**152**, Figure 8). The new compound, a meroditerpene, sartorypyrone C (**152**), was obtained from a rare sponge-derived fungus *Neosartorya paulistensis*. The antibacterial activity of sartorypyrone C against four reference strains (*Staphylococcus aureus, Bacillus subtilis, Escherichia coli,* and *Pseudomonas aeruginosa*) was tested, but no significant activity was observed [67].

### 3.12. Penicillium

As an important source of bioactive secondary metabolites, *Penicillium* produced many diterpenes with novel structures [10]. From 2009 to 2021, 15 articles reported the discovery of 45 new diterpenes (**153**–**197**, Figure 8) from marine *Penicillium*.

Six new diterpenes, named conidiogenones B–G (**153**–**158**), were obtained from a deep-sea sediment-derived fungus *Penicillium* sp. F23-2 [27]. Their cytotoxic activities were evaluated on HL-60, A-549, BEL-7402, and MOLT-4 cell lines. To the A-549 cell line, compounds **153** and **157** showed weak cytotoxicity with IC_50_ values of 40.3 and 42.2 µM, respectively, while compounds **155**, **156**, and **158** displayed much stronger cytotoxicity with IC_50_ values of 9.3, 15.1, and 8.3 µM, respectively. To the HL-60 cell line, compounds **153** and **157** showed weak cytotoxicity with IC_50_ values of 28.2 and 17.8 µM, respectively, while compounds **155**, **156**, and **158** exhibited much stronger cytotoxicity with IC_50_ values of 5.3, 8.5, and 1.1 µM, respectively. Compound **154** showed ultra-high activity against the HL-60 cell line with an IC_50_ value of 0.038 µM. In addition, it also displayed ultra-high activity against the BEL-7402 cell line with an IC_50_ value of 0.97 µM, while compounds **155**, **157**, and **158** showed moderate to weak activities against the same cell line with IC_50_ values of 11.7, 17.1, and 43.2 µM, respectively. Only compounds **155**, **157**, and **158** showed biological activities against the MOLT-4 cell line with IC_50_ values of 21.1, 25.8, and 4.7 µM, respectively.

By activity tracing, Gao et al. isolated two unusual diterpenes cyclopiasconidiogenones H and I (**159** and **160**) from a red alga-derived fungus *Penicillium chrysogenum* QEN-24S [68]. However, the two compounds did not show biological activity in the antimicrobial test.

Six novel indole diterpenoids (**161**–**166**) were obtained from a mangrove-derived fungus *Penicillium camemberti* OUCMDZ-1492 [47]. Among them, compounds **161**–**163** and **165** exhibited weak activities against the H1N1 virus, with IC_50_ values of 28.3, 38.9, 32.2, and 73.3 µM, respectively.

A novel spirotetracyclic diterpene with a 5/5/5/5 spiro-carbon skeleton structure, named spirograterpene A (**167**), was obtained from the deep-sea fungus *Penicillium granulatum* MCCC 3A00475 [23]. Spirograterpene A showed anti-allergic effects on immunoglobulin E (IgE)-mediated rat mast RBL-2H3 cells. Its inhibition rate was 18% at 20 µg/mL. This data of loratadine serving as a positive control was 35% at the same concentration.

Three new indole diterpenes, 22-hydroxylshearinine F (**168**), 6-hydroxylaspalinine (**169**), and 7-O-acetylemindole SB (**170**), were obtained from a sea-anemone-derived fungus, *Penicillium* sp. AS-79 [38]. Among them, compound **169** was active against the aquatic pathogen *Vibrio parahaemolyticus* with an MIC of 64.0 µg/mL, compared with 0.5 µg/mL for the positive control chloromycetin.

Moreover, three new cyclopiane diterpenes (**161**–**173**) were isolated from a deep-sea fungus *Penicillium commune* MCCC 3A00940. They all contain a rigid 6/5/5/5 fused tetracyclic ring framework, which is rare in nature [69].

Cheng et al. also isolated three cyclopiane diterpenes (**174**–**176**) from a deep-sea sediment-derived fungus *Penicillium* sp. YPGA11 [70]. The compound conidiogenol D (**175**) showed weak cytotoxic activity against five esophageal cancer cell lines (EC109, KYSE70, EC9706, KYSE30, and KYSE450) with IC_50_ values ranging from 25 to 55 µM.

Furthermore, 15 indole diterpenes (**177**–**191**) were successively obtained from the *Penicillium* sp. KFD28, a fungus derived from bivalve mollusk [28,44,45,71]. Compound **178** represents the first indole diterpenoid with a unique pyridine-containing heptacyclic ring system. Compound **181** is an indole diterpenoid with a unique 6/5/5/6/6/5/5 heptacyclic system. Compound **183** contains an additional oxygen atom between C-21 and C-22 compared to paxilline, and thus, forms an unusual 6/5/5/6/6/7 hexacyclic ring system bearing a 1,3-dioxepane ring, which is rarely encountered in natural products. Compounds **177**, **178, 181**, **182**, **184**, **185**, and **190** showed potent inhibitory activities against protein tyrosine phosphatase (PTP1B) with IC_50_ values of 1.7, 2.4, 14, 27, 23, 31.5, and 9.5 µM, respectively, compared with 1.6 µM for the positive control, Na_3_VO_4_. Compound **189** had a weak activity against HeLa cells with an IC_50_ value of 36.3 µM, whereas the value for the positive control, cisplatin, was 8.6 µM.

An indole diterpene, named penicindopene A (**192**), was obtained from the fungus *Penicillium* sp. YPCMAC1, collected at a depth of −4500 m in the western Pacific Ocean [29]. This is the first report of indole diterpenes containing a 3-hydroxy-2-indolone moiety. Penicindopene A also showed moderate cytotoxic activities against A-549 and HeLa cell lines with IC_50_ values of 15.2 and 20.5 µM, respectively.

In addition, *Penicillium thomii* YPGA3, *Penicillium* sp. YPGA11, and *Penicillium* sp. YPCMAC1 were all derived from the deep-sea water at a depth of −4500 m in the Yap Trench (West Pacific Ocean). A rare 19-nor labdane-type diterpenoid, named penitholabene (**193**), was isolated from *Penicillium thomii* YPGA3 [46]. This represents the first 19-nor labdane-type diterpenoid found in nature. It showed an inhibitory effect against α-glucosidase with an IC_50_ value of 282 µM, being more active than the positive control, acarbose (1330 µM).

A new pimarane diterpene, named diaporthein C (**194**), was obtained from a sea slug gut-derived fungus, *Penicillium sclerotiorum* GZU-XW03-2 [72]. This is the third pimarane diterpene identified with a Δ^8(9)^ double bond.

Three unreported cyclopiane diterpenes (**195**–**197**) were obtained from the deep-sea sediment fungus *Penicillium* sp. TJ403-2 [73]. The anti-inflammatory activities of these compounds were evaluated. Compound **195** could significantly reduce LPS-induced NO production with an IC_50_ value of 2.19 mM, which was only one-third of that of the positive control, indomethacin.

### 3.13. Stachybotrys

**Figure 9:**
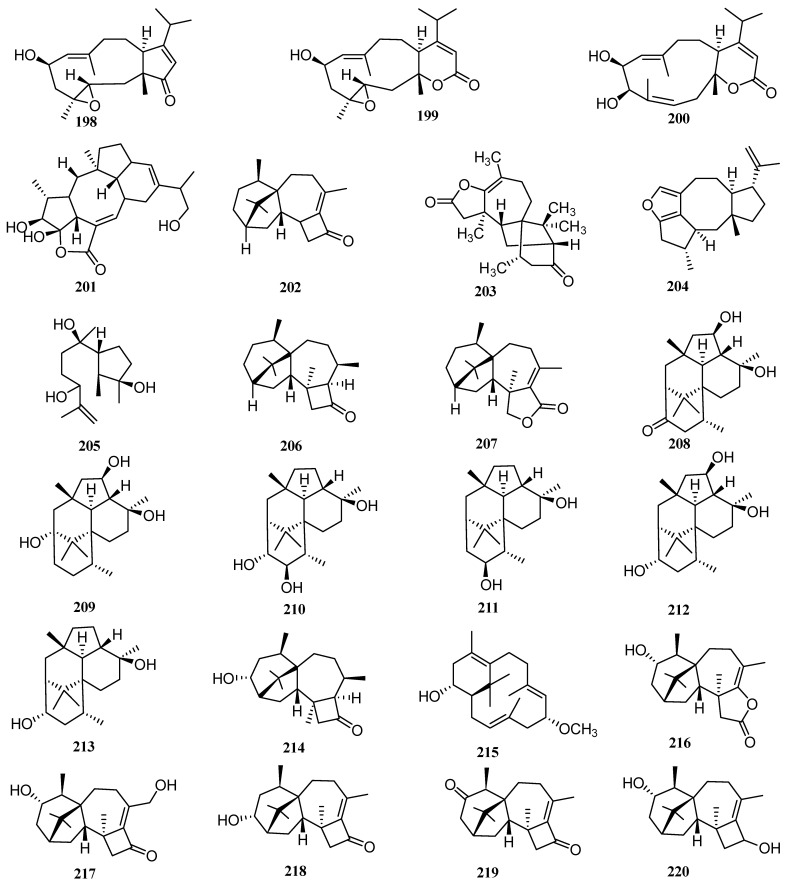

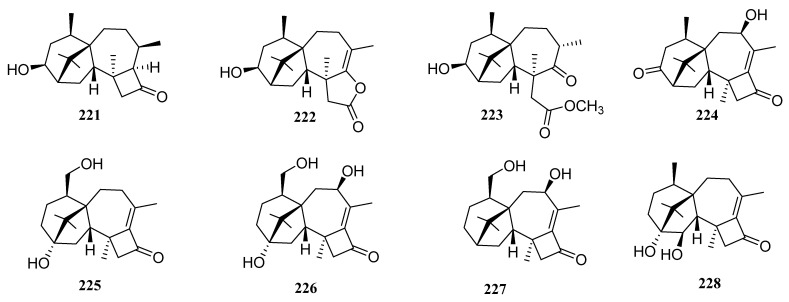
Chemical structures of diterpenes (**198**–**200** from *Stachybotrys* sp., **201** from *Talaromyces* sp., **202**–**228** from *Trichoderma* sp.).

Three new diterpenes were generated by the genus *Stachybotrys* (**198**–**200**, Figure 9). These compounds, named stachatranones A–C (**198**–**200**), are all of the dolabelllane-type. They were isolated from a coral-derived fungus *Stachybotrys chartarum* TJ403-SS6 [39]. Stachatranone B exhibited an inhibitory effect on *Acinetobacter baumannii* with an MIC value of 16 µg/mL, compared with 2 and 8 µg/mL for the positive controls, amikacin and vancomycin, respectively. Stachatranone B also showed an inhibitory effect on *Enterococcus faecalis* with an MIC value of 32 µg/mL, compared with 0.5 µg/mL for vancomycin.

### 3.14. Talaromyces

Only one new diterpene was produced by the genus *Talaromyces* (**201**, Figure 9). The compound, roussoellol C (**201**), was obtained from the fungus *Talaromyces purpurogenus* PP-414 isolated from a beach in Qinhuangdao, Hebei Province [74]. It was cytotoxic to MCF-7 cells with an IC_50_ of 6.5 µM.

### 3.15. Trichoderma

Marine-derived fungi of the *Trichoderma* genus have produced many structurally novel natural products with diverse bioactivities [75]. From 2009 to 2021, 11 articles reported the discovery of 27 new diterpenes (**202**–**228**, Figure 9) from marine *Trichoderma*.

A new harziane dieterpene harzianone (**202**) was isolated from a seaweed endophytic fungus *Trichoderma longibrachiatum* [40]. It displayed antibacterial effects on *Escherichia coli* and *Staphylococcus aureus* at 30 µg/disk (inhibitory diameters of 8.3 and 7.0 mm, respectively), while chloramphenicol as the positive control showed inhibitory diameters of 22 mm at 20 µg/disc. In addition, harzianone showed 82.6% of lethality in brine shrimp (*Artemia salina* L.) larvae at 100 µg/mL.

Xie et al. detected unusual signals in the ^13^C NMR spectra recorded on the fractions of the fungus *Trichoderma erinaceum* [76], and thereafter identified a new diterpene trichodermaerin (**203**) in the subsequent fermentation and isolation.

A novel diterpene trichocitrin (**204**) was isolated from the culture of the fungus *Trichoderma citrinoviride* cf-27 isolated from the seaweed surface. This represents both the first report of the isolation of a fusicoccane diterpene from *Trichoderma*, and the first discovery of a furan-bearing fusicoccane diterpene. At 20 µg/disk, trichocitrin formed an 8.0 mm inhibition zone against *Escherichia coli* [41]. Later, a fungus, *Trichoderma asperellum* cf44-2, was isolated from the alga collected in the same batch. Additionally, an unreported diterpene, named 11-hydroxy-9-harzien-3-one (**205**), was isolated from the fermentation of this fungus [77].

Two harziane diterpenoids (**206**–**207**) were isolated from a mangrove-derived fungus, *Trichoderma* sp. Xy24 [31]. Compound **206** exhibited low cytotoxic activities against the HeLa and MCF-7 cell lines with IC_50_ values of 30.1 and 30.7 mM, respectively.

Six new diterpenes, trichodermanins C–H (**208**–**213**), with a rare fused 6/5/6/6 ring system, were isolated from a sponge-derived fungus *Trichoderma harzianum* OUPS-111D-4 [32,78]. Trichodermanin C had significant cytotoxic activities against P388, HL-60, and L1210 cell lines with IC_50_ values of 7.9, 6.8, and 7.6 µM, respectively, compared with 6.1, 5.1, and 4.5 µM for the positive control, 5-fluorouraci. Compound **211** showed weak cytotoxic activities against these cell lines with IC_50_ values exceeding 40 µM.

Two new diterpenes, named 3*R*-hydroxy-9*R*,10*R*-dihydroharzianone (**214**), and 11*R*-methoxy-5,9,13-proharzitrien-3-ol (**215**), were isolated from the fungus *Trichoderma harzianum* X-5 derived from the surface of a brown alga *Laminaria japonica* [52]. Among them, compound **215** has a bicyclic skeleton that is rarely reported. The growth-inhibitory effects of these two compounds were tested on four phytoplankton species, *Chattonella marina*, *Heterosigma akashiwo*, *Karlodinium veneficum*, and *Prorocentrum donghaiense*. Compound **214** had inhibitory activity against *Chattonella marina* with an IC_50_ value of 7.0 µg/mL. Compound **215** displayed an excellent inhibitory effect on the growth of all four kinds of phytoplankton, with IC_50_ values of 1.2, 1.3, 3.2, and 4.3 µg/mL, respectively, compared with 0.46, 0.98, 0.89, and 1.9 µM for the positive control, K_2_Cr_2_O_7_.

Five new diterpenes, named harzianones A–D (**216**–**219**) and harziane (**220**), were isolated from a soft coral-derived fungus, *Trichoderma harzianum* XS 20,090,075 [48]. Compounds **216**–**220** were extremely phytotoxic, inhibiting the germination of amaranth and lettuce seeds at a concentration of 200 ppm. This is the first time that phytotoxic compounds were isolated from *Trichoderma*.

Three new harziane derivatives 3*S*-hydroxy-9*R*,10*R*-dihydroharzianone, 3*S*-hydroxytrichodermaerin, and methyl 3*S*-hydroxy-10,11-seco-harzianate (**221**–**223**) were isolated from an algicolous fungus, *Trichoderma asperelloides* RR-dl-6-11 [79]. This is the first work reporting secondary metabolites of *Trichoderma asperelloides*. The compounds were tested for inhibitory activity against four marine bacteria *Vibrio anguillarum*, *V. harveyi*, *V. parahemolyticus*, and *V. splendidus*. At 100 µg/disc, compounds **221**–**223** did not show any observable inhibitory effect against any of the tested marine bacteria.

Five new harziane-type diterpenes named harzianols K–O (**224**–**228**) were obtained from a deep-sea sediment-derived fungus, *Trichoderma* sp. SCSIOW21 [54]. Compound **225** had a strong anti-inflammatory effect. It showed a NO inhibition rate of 81.8% at 100 µM.

### 3.16. Others

**Figure 10:**
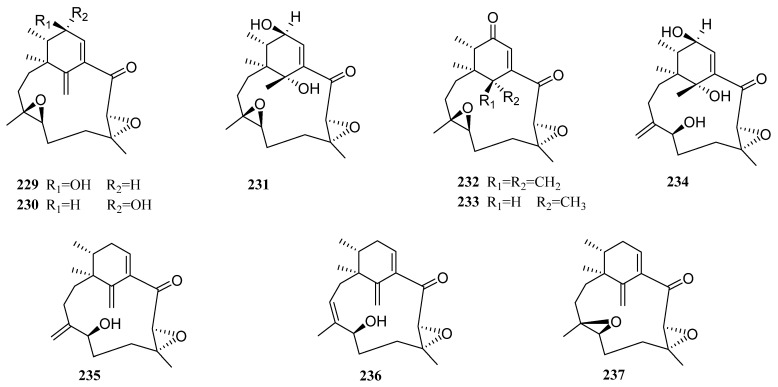
Chemical structures of diterpenes (**229**–**237** from unidentified fungus).

In addition to the 15 genera mentioned above, there are also some marine fungi whose taxonomic status has not been determined, but their secondary metabolites have been obtained and studied. Nine new diterpenoids were reported to be produced by unidentified marine fungi (**229**–**237**, Figure 10). They were named phomactin I (**229**), 13-epi-phomactin I (**230**), phomactin J (**231**), phomactins K–M (**232**–**234**), and phomactins N−P (**235**–**237**). They were isolated by Masahiro Ishino et al. from a fungus of unknown red algal origin [80,81,82]. HUVECs, NHDF (normal human dermal fibroblasts) cells, and HeLa cells were used to test the cytotoxicity of these compounds. However, they did not show any observable cytotoxic effect.

## 4. Conclusions

This review provides a comprehensive overview of the structures and activities of 237 new diterpenes discovered from 47 strains of marine-derived fungi from 2009 to 2021. The articles reporting *Penicillium*, *Aspergillus*, and *Trichoderma* accounted for the majority (64%) of all the relevant publications. The numbers of diterpenes isolated from the four genera *Botryotinia* (80), *Penicillium* (45), *Aspergillus* (38), and *Trichoderma* (27) are the top four. It is noteworthy that 80 new diterpenes were isolated from a single strain of the genus *Botryotinia*, 71 of which are aphidicolin congeners. After aphidicolanes, indole-type diterpenes (46) are the most numerous diterpenes, followed by pimarane-type (29), harziane-type (16), and cyclopiane-type (9) diterpenes. Among the bioactive compounds, the compounds with cytotoxic activity were the most, accounting for 36%, followed by compounds with antibacterial effects, accounting for 20%. The compound with the most notable cytotoxicity is conidiogenone C (**154**), which showed cytotoxic activities in HL-60 and BEL-7402 cell lines, with IC_50_ values of 0.038 and 0.97 µM, respectively. The compound with the most promising antimicrobial activity is aspewentin D (**12**). It showed inhibitory activity against *Edwardsiella tarda* and *Vibrio harveyi* with MIC values of 2.0 and 4.0 µg/mL, respectively. These marine-derived diterpenes show rich structural diversities and bioactivities. The reported compounds partially uncovered the untapped potential of marine fungi as diterpene producers.

## Figures and Tables

**Table 1:** Marine fungi-derived compounds with various bioactivities.

Compound Number	Compound Name	Producing Organism	Activity	Reference
			Cytotoxicity to cancer cell lines	
**31**	Ascandinine D	*Aspergillus candidus* HDN15-152	Cytotoxicity against HL-60 cells with an IC_50_ value of 7.8 µM	[18]
**38**	(2*R*,4b*R*,6a*S*,12b*S*,12c*S*,14a*S*)-4b-Deoxy-*β*-aflatrem	*Aspergillus flavus* OUCMDZ-2205	Activity to A-549 cell cycle in the S phase with an IC_50_ value of 10 µM	[19]
**39**	(2*R*,4b*S*,6a*S*,12b*S*,12c*R*)-9-Isopentenylpaxilline D	*Aspergillus flavus* OUCMDZ-2205	Activity to A-549 cell cycle in the S phase with an IC_50_ value of 10 µM	[19]
**46**	Anthcolorin H	*Aspergillus versicolor*	Activity to Hela cells with an IC_50_ value of 43.7 µM	[20]
**56**	Aphidicolin A8	*Botryotinia fuckeliana* MCCC 3A00494	Activity to T24 and HL-60 cells with IC_50_ values of 2.5 and 6.1 µM, respectively	[21]
**129**	Penicilindole A	*Eupenicillium* sp. HJ002	Activity to A-549 and HepG2 cell lines with IC_50_ values of 5.5 and 1.5 µM, respectively	[22]
**132**	Scopararane C	*Eutypella scoparia* FS26	Cytotoxicity against the MCF-7 cell line with an IC_50_ value of 35.9 µM	[23]
**133**	Scopararane D	*Eutypella scoparia* FS26	Cytotoxicity against the MCF-7 cell line with an IC_50_ value of 25.6 µM	[23]
**138**	Scopararane I	*Eutypella* sp. FS46	Inhibitory activities against NCI-H460 and SF-268 cell lines with IC_50_ values of 13.59 and 25.31 µg/mL, respectively	[24]
**139**	Aspergilone A	*Epicoccum* sp. HS-1	Inhibits the growth of human epidermis carcinoma cell line and multidrug-resistant cell line expressing high levels of P-gp with IC_50_ values of 3.51 and 2.34 µg/mL, respectively	[25]
**140**	Aspergilone B ^1^	*Epicoccum* sp. HS-1	Inhibits the growth of human epidermis carcinoma cell line and multidrug-resistant cell line expressing high levels of P-gp with IC_50_ values of 20.74 and 14.47 µg/mL, respectively	[25]
**146**	Rhizovarin A	*Mucor irregularis* QEN-189	Activity against A-549 and HL-60 cancer cell lines with IC_50_ values of 11.5 and 9.6 µM, respectively	[26]
**147**	Rhizovarin B	*Mucor irregularis* QEN-189	Activity against A-549 and HL-60 cancer cell lines with IC_50_ values of 6.3 and 5.0 µM, respectively	[26]
**151**	Rhizovarin F	*Mucor irregularis* QEN-189	Activity against the A-549 cancer cell line with an IC_50_ value of 9.2 µM	[26]
**153**	Conidiogenone B	*Penicillium* sp. F23-2	Cytotoxicity to A-549 and HL-60 cell lines with IC_50_ values of 40.3 and 28.2 µM, respectively	[27]
**154**	Conidiogenone C	*Penicillium* sp. F23-2	Activity to HL-60 and BEL-7402 cell lines with IC_50_ values of 0.038 and 0.97 µM, respectively	[27]
**155**	Conidiogenone D	*Penicillium* sp. F23-2	Activity to A-549, HL-60, BEL-7402, and MOLT-4 cell lines with IC_50_ values of 9.3, 5.3, 11.7, and 21.1 µM, respectively	[27]
**156**	Conidiogenone E	*Penicillium* sp. F23-2	Activity to A-549 and HL-60 cell lines with IC_50_ values of 15.1 and 8.5 µM, respectively	[27]
**157**	Cnidiogenone F	*Penicillium* sp. F23-2	Activity to A-549, HL-60, BEL-7402, and MOLT-4 cell lines with IC_50_ values of 42.2, 17.8, 17.1, and 25.8 µM, respectively	[27]
**158**	Conidiogenone G	*Penicillium* sp. F23-2	Activity to A-549, HL-60, BEL-7402, and MOLT-4 cell lines with IC_50_ values of 8.3, 1.1, 43.2, and 4.7 µM, respectively	[27]
**189**	Penerpene M	*Penicillium* sp. KFD28	Activity against HeLa cells with an IC_50_ value of 36.3 µM	[28]
**192**	Penicindopene A	*Penicillium* sp. YPCMAC1	Cytotoxicity against A-549 and HeLa cell lines with IC_50_ values of 15.2 and 20.5 µM, respectively	[29]
**201**	Roussoellol C	*Talaromyces purpurogenus* PP-414	Cytotoxic to MCF-7 cells with an IC_50_ value of 6.5 µM	[30]
**206**	(9*R*,10*R*)-dihydro-harzianone	*Trichoderma* sp. Xy24	Activity against HeLa and MCF-7 cell lines with IC_50_ values of 30.1 and 30.7 µM, respectively	[31]
**208**	Trichodermanin C	*Trichoderma harzianum* OUPS-111D-4	Cytotoxic activity against P388, HL-60, and L1210 cell lines with IC_50_ values of 7.9, 6.8, and 7.6 µM, respectively	[32]
			Antimicrobial activity	
**10**	Asperolide D	*Aspergillus wentii* SD-310	Inhibits *Edwardsiella tarda* with an MIC value of 16 µg/mL	[33]
**12**	Aspewentin D	*Aspergillus wentii* SD-310	Activity against *Fusarium graminearum* and *Micrococcus luteus*, with MIC values of 2.0 and 4.0 µg/mL, respectively	[34]
**14**	Aspewentin F	*Aspergillus wentii* SD-310	Inhibitory activity against *Edwardsiella tarda* and *Vibrio harveyi* with MIC values of 4.0 and 8.0 µg/mL, respectively	[34]
**15**	Aspewentin G	*Aspergillus wentii* SD-310	Inhibitory activity against *Vibrio harveyi* with an MIC value of 4.0 µg/mL	[34]
**16**	Aspewentin H	*Aspergillus wentii* SD-310	Activity against *Pseudomonas aeruginosa* and *Fusarium graminearum* with MIC values of 4.0 and 4.0 µg/mL, respectively	[34]
**43**	19-hydroxypenitrem A	*Aspergillus nidulans* EN-330	Activity against pathogens *Edwardsiella tarda*, *Vibrio anguillarum*, *Escherichia coli,* and *Staphylococcus aureus* with MIC values of 16, 32, 16, and 16 µg/mL, respectively	[35]
**47**	(3*R*,9*S*,12*R*,13*S*,17*S*,18*S*)-2-carbonyl-3-hydroxylemeniveol	*Aspergillus versicolor* ZZ761	Antimicrobial activity against *Escherichia coli* and *Candida albicans* with MIC values of 20.6 and 22.8 µM, respectively	[36]
**145**	Micromonohalimane B	*Micromonospora* sp.	Inhibitory effect on methicillin-resistant *Staphylococcus aureus* with an MIC value of 40 µg/mL	[37]
**169**	6-hydroxylpaspalinine	*Penicillium* sp. AS-79	Activity against the aquatic pathogen *Vibrio parahaemolyticus* with an MIC value of 64.0 µg/mL	[38]
**199**	Stachatranone B	*Stachybotrys chartarum* TJ403-SS6	Activity against *Acinetobacter baumannii* and *Enterococcus faecalis* with MIC values of 16 and 32 µg/mL	[39]
**202**	Harzianone	*Trichoderma longibrachiatum*	Effect on *Escherichia coli* and *Staphylococcus aureus* at 30 µg/disk (with inhibitory diameters of 8.3 and 7.0 mm, respectively)	[40]
**204**	Trichocitrin	*Trichoderma citrinoviride* cf-27	Inhibit *Escherichia coli* with an inhibitory diameter of 8.0 mm at 20 µg/disk	[41]
**17**–**18**	Aspewentins I–J	*Aspergillus wentii* SD-310	Inhibitory activity against *Edwardsiella tarda, Vibrio harveyi,* and *V. parahemolyticus*, each with an MIC value of 8.0 µg/mL	[42]
			Inhibition of enzymes	
**38**	(2*R*,4b*R*,6a*S*,12b*S*,12c*S*,14a*S*)-4b-Deoxy-*β*-aflatrem	*Aspergillus flavus* OUCMDZ-2205	Inhibitory effect on the kinase PKC-*β* with an IC_50_ value of 15.6 µM	[19]
**142**	Isopimarane diterpene	*Epicoccum* sp. HS-1	Inhibits α-glucosidase with an IC_50_ value of 4.6 µM	[43]
**177**–**178**	Penerpenes A–B	*Penicillium* sp. KFD28	Inhibitory activity against protein tyrosine phosphatase (PTP1B) with IC_50_ values of 1.7 and 2.4 µM, respectively	[44]
**181**–**182**	Penerpenes E–F	*Penicillium* sp. KFD28	Inhibitory activity against PTP1B with an IC_50_ value of 14 and 27 µM, respectively	[45]
**184**–**185**	Penerpenes H–I	*Penicillium* sp. KFD28	Inhibitory activity against PTP1B with an IC_50_ value of 23 and 31.5 µM, respectively	[45]
**190**	Penerpene N	*Penicillium* sp. KFD28	Inhibitory activity against PTP1B with an IC_50_ value of 9.5 µM	[28]
**193**	Penitholabene	*Penicillium thomii* YPGA3	Inhibitory effect against α-glucosidase with an IC_50_ value of 282 µM	[46]
			Antivirus	
**30**	Ascandinine C	*Aspergillus candidus* HDN15-152	Anti-influenza virus A (H1N1) activity with an IC_50_ value of 26 µM	[18]
**161**	3-deoxo-4b-deoxypaxilline	*Penicillium camemberti* OUCMDZ-1492	Activity against the H1N1 virus with an IC_50_ value of 28.3 µM	[47]
**162**	4a-demethylpaspaline-4a-carboxylic acid	*Penicillium camemberti* OUCMDZ-1492	Activity against the H1N1 virus with an IC_50_ value of 38.9 µM	[47]
**163**	4a-demethylpaspaline-3,4,4a-triol	*Penicillium camemberti* OUCMDZ-1492	Activity against the H1N1 virus with an IC_50_ value of 32.2 µM	[47]
**165**	9,10-diisopentenylpaxilline	*Penicillium camemberti* OUCMDZ-1492	Activity against the H1N1 virus with an IC_50_ value of 73.3 µM	[47]
			Inhibition of the germination of seeds	
**216−219**	Harzianones A−D	*Trichoderma harzianum* XS 20090075	Inhibits the germination of amaranth and lettuce seeds at a concentration of 200 ppm	[48]
**220**	Harziane	Trichoderma harzianum XS 20090075	Inhibiting the germination of amaranth and lettuce seeds at a concentration of 200 ppm	[48]
			Others	
**1**	JBIR-65	*Actinomadura* sp. SpB081030SC-15	Protects neuronal hybridoma N18-RE-105 cells with an EC_50_ value of 31 µM	[49]
**5**	Arthrinin D	*Arthrinium* sp.	Inhibits VEGF-A (vascular endothelial growth factor A)-dependent endothelial cell sprouting with an IC_50_ of 2.6 µM	[50]
**6**	Myrocin D	*Arthrinium* sp.	Inhibits VEGF-A-dependent endothelial cell sprouting with an IC_50_ of 3.7 µM	[50]
**35**	Aspewentin A	*Aspergillus wentii* na-3	Active against *Chattonella marina* and *Heterosigma akashiwo*, with LC_50_ values of 0.81 and 2.88 µM, respectively	[51]
**214**	3*R*-hydroxy-9*R*,10*R*-dihydroharzianone	*Trichoderma harzianum* X-5	Activity against *Chattonella marina* with an IC_50_ value of 7.0 µg/mL	[52]
**215**	11*R*-methoxy-5,9,13-proharzitrien-3-ol	*Trichoderma harzianum* X-5	Inhibitory effect on the growth of four kinds of phytoplankton *Chattonella marina*, *Heterosigma akashiwo*, *Karlodinium veneficum*, and *Prorocentrum donghaiense* with IC_50_ values of 1.2, 1.3, 3.2, and 4.3 µg/mL, respectively	[52]
**36**	Aspewentin B	*Aspergillus wentii* na-3	Inhibits the growth of *Artemia salina* with an LC_50_ value of 6.36 µM	[51]
**120**	Botryotin A	*Botryotinia fuckeliana* MCCC 3A00494	Anti-allergic activity with an IC_50_ value of 0.2 mM	[53]
**167**	Spirograterpene A	*Penicillium granulatum* MCCC 3A00475	Anti-allergic effect on immunoglobulin E (IgE)-mediated rat mast RBL-2H3 cells with 18% inhibition at 20 µg/mL	[23]
**202**	Harzianone	*Trichoderma longibrachiatum*	82.6% of lethality to brine shrimp (*Artemia salina* L.) larvae at 100 µg/mL	[40]
**225**	Harzianol L	*Trichoderma* sp. SCSIOW21	Anti-inflammatory effect with 81.8% NO inhibition at 100 µM	[54]

^1^ Compounds **139**−**141** were reported as compounds 1−3 in the reference [25], where only compound **139** (numbered as compound 1 in the reference) was named as aspergilone A, while the names for compounds **140** and **141** (numbered as compounds 2 and 3, respectively, in the reference) were not provided. The name aspergilone B was used to represent compound **140** in this article.

## Data Availability

Not applicable.

## Abbreviations

HDAC, Histone deacetylase; HUVEC, Human umbilical vascular endothelial cells; LC-MS, Liquid chromatography-mass spectrometry; NHDF, Normal human dermal fibroblasts; NMR, Nuclear magnetic resonance; PKC, Protein kinase C; RBL, Rat basophilic leukemia; SAHA, Suberoylanilide hydroxamic acid; VEGF-A, Vascular endothelial growth factor A.

## References

[1] Blunt J.W., Copp B.R., Keyzers R.A., Munro M.H., Prinsep M.R. (2014). Marine natural products. Nat. Prod. Rep..

[2] Carroll A.R., Copp B.R., Davis R.A., Keyzers R.A., Prinsep M.R. (2021). Marine natural products. Nat. Prod. Rep..

[3] Blunt J.W., Copp B.R., Keyzers R.A., Munro M.H.G., Prinsep M.R. (2017). Marine natural products. Nat. Prod. Rep..

[4] Blunt J.W., Carroll A.R., Copp B.R., Davis R.A., Keyzers R.A., Prinsep M.R. (2018). Marine natural products. Nat. Prod. Rep..

[5] Carroll A.R., Copp B.R., Davis R.A., Keyzers R.A., Prinsep M.R. (2019). Marine natural products. Nat. Prod. Rep..

[6] Carroll A.R., Copp B.R., Davis R.A., Keyzers R.A., Prinsep M.R. (2020). Marine natural products. Nat. Prod. Rep..

[7] Blunt J.W., Copp B.R., Keyzers R.A., Munro M.H., Prinsep M.R. (2015). Marine natural products. Nat. Prod. Rep..

[8] Blunt J.W., Copp B.R., Keyzers R.A., Munro M.H., Prinsep M.R. (2016). Marine natural products. Nat. Prod. Rep..

[9] Willems T., De Mol M.L., De Bruycker A., De Maeseneire S.L., Soetaert W.K. (2020). Alkaloids from Marine Fungi: Promising Antimicrobials. Antibiotics.

[10] Jiang M., Wu Z., Guo H., Liu L., Chen S. (2020). A Review of Terpenes from Marine-Derived Fungi: 2015–2019. Mar. Drugs.

[11] Shabana S., Lakshmi K.R., Satya A.K. (2021). An Updated Review of Secondary Metabolites from Marine Fungi. Mini Rev. Med. Chem..

[12] Youssef F.S., Ashour M.L., Singab A.N.B., Wink M. (2019). A Comprehensive Review of Bioactive Peptides from Marine Fungi and Their Biological Significance. Mar. Drugs.

[13] Huang M., Lu J.J., Huang M.Q., Bao J.L., Chen X.P., Wang Y.T. (2012). Terpenoids: Natural products for cancer therapy. Expert Opin. Investig. Drugs.

[14] Hanson J.R. (1986). Diterpenoids. Nat. Prod. Rep..

[15] Hanson J.R. (2009). Diterpenoids. Nat. Prod. Rep..

[16] Hanson J.R. (2011). Diterpenoids of terrestrial origin. Nat. Prod. Rep..

[17] Hanson J.R., Nichols T., Mukhrish Y., Bagley M.C. (2019). Diterpenoids of terrestrial origin. Nat. Prod. Rep..

[18] Takagi M., Motohashi K., Khan S.T., Hashimoto J., Shin-Ya K. (2010). JBIR-65, a new diterpene, isolated from a sponge-derived *Actinomadura* sp. SpB081030SC-15. J. Antibiot..

[19] Zhou G., Sun C., Hou X., Che Q., Zhang G., Gu Q., Liu C., Zhu T., Li D. (2021). Ascandinines A-D, indole diterpenoids, from the sponge-derived fungus *Aspergillus candidus* HDN15-152. J. Org. Chem..

[20] Sun K., Li Y., Guo L., Wang Y., Liu P., Zhu W. (2014). Indole diterpenoids and isocoumarin from the fungus, *Aspergillus flavus*, isolated from the prawn, *Penaeus vannamei*. Mar. Drugs.

[21] Elsbaey M., Tanaka C., Miyamoto T. (2019). New secondary metabolites from the mangrove endophytic fungus *Aspergillus versicolor*. Phytochem. Lett..

[22] Niu S., Xia J.M., Li Z., Yang L.H., Yi Z.W., Xie C.L., Peng G., Luo Z.H., Shao Z., Yang X.W. (2019). Aphidicolin chemistry of the deep-sea-derived fungus *Botryotinia fuckeliana* MCCC 3A00494. J. Nat. Prod..

[23] Zheng C.J., Bai M., Zhou X.M., Huang G.L., Shao T.M., Luo Y.P., Niu Z.G., Niu Y.Y., Chen G.Y., Han C.R. (2018). Penicilindoles A-C, cytotoxic indole diterpenes from the mangrove-derived fungus *Eupenicillium* sp. HJ002. J. Nat. Prod..

[24] Niu S., Fan Z.W., Xie C.L., Liu Q., Luo Z.H., Liu G., Yang X.W. (2017). Spirograterpene A, a tetracyclic spiro-diterpene with a fused 5/5/5/5 ring system from the deep-sea-derived fungus *Penicillium granulatum* MCCC 3A00475. J. Nat. Prod..

[25] Liu H., Zhang L., Chen Y., Li S., Tan G., Sun Z., Pan Q., Ye W., Li H., Zhang W. (2017). Cytotoxic pimarane-type diterpenes from the marine sediment-derived fungus *Eutypella* sp. FS46. Nat. Prod. Res..

[26] Xia X., Zhang J., Zhang Y., Wei F., Liu X., Jia A., Liu C., Li W., She Z., Lin Y. (2012). Pimarane diterpenes from the fungus *Epicoccum* sp. HS-1 associated with *Apostichopus japonicus*. Bioorg. Med. Chem. Lett..

[27] Gao S.S., Li X.M., Williams K., Proksch P., Ji N.Y., Wang B.G. (2016). Rhizovarins A-F, indole-diterpenes from the mangrove-derived endophytic fungus *Mucor irregularis* QEN-189. J. Nat. Prod..

[28] Du L., Li D., Zhu T., Cai S., Wang F., Xiao X., Gu Q. (2009). New alkaloids and diterpenes from a deep ocean sediment derived fungus *Penicillium* sp.. Tetrahedron.

[29] Dai L.T., Yang L., Kong F.D., Ma Q.Y., Xie Q.Y., Dai H.F., Yu Z.F., Zhao Y.X. (2021). Cytotoxic indole-diterpenoids from the marine-derived fungus *Penicillium* sp. KFD28. Mar. Drugs.

[30] Liu L., Xu W., Li S., Chen M., Cheng Y., Yuan W., Cheng Z., Li Q. (2019). Penicindopene A, a new indole diterpene from the deep-sea fungus *Penicillium* sp. YPCMAC1. Nat. Prod. Res..

[31] Zhao W.Y., Yi J., Chang Y.B., Sun C.P., Ma X.C. (2022). Recent studies on terpenoids in Aspergillus fungi: Chemical diversity, biosynthesis, and bioactivity. Phytochemistry.

[32] Zhang M., Liu J.-M., Zhao J.-L., Li N., Chen R.-D., Xie K.-B., Zhang W.-J., Feng K.-P., Yan Z., Wang N. (2016). Two new diterpenoids from the endophytic fungus *Trichoderma* sp. Xy24 isolated from mangrove plant *Xylocarpus granatum*. Chin. Chem. Lett..

[33] Yamada T., Suzue M., Arai T., Kikuchi T., Tanaka R. (2017). Trichodermanins C-E, new diterpenes with a fused 6-5-6-6 ring system produced by a marine sponge-derived fungus. Mar. Drugs.

[34] Li X.D., Li X., Li X.M., Xu G.M., Zhang P., Meng L.H., Wang B.G. (2016). Tetranorlabdane diterpenoids from the deep sea sediment-derived fungus *Aspergillus wentii* SD-310. Planta Med..

[35] Li X.D., Li X.M., Li X., Xu G.M., Liu Y., Wang B.G. (2016). Aspewentins D-H, 20-nor-isopimarane derivatives from the deep sea sediment-derived fungus *Aspergillus wentii* SD-310. J. Nat. Prod..

[36] Zhang P., Li X.-M., Li X., Wang B.-G. (2015). New indole-diterpenoids from the algal-associated fungus *Aspergillus nidulans*. Phytochem. Lett..

[37] Zhang D., Yi W., Ge H., Zhang Z., Wu B. (2021). A new antimicrobial indoloditerpene from a marine-sourced fungus *Aspergillus versicolor* ZZ761. Nat. Prod. Res..

[38] Zhang Y., Adnani N., Braun D.R., Ellis G.A., Barns K.J., Parker-Nance S., Guzei I.A., Bugni T.S. (2016). Micromonohalimanes A and B: Antibacterial halimane-type diterpenoids from a marine *Micromonospora* species. J. Nat. Prod..

[39] Hu X.Y., Meng L.H., Li X., Yang S.Q., Li X.M., Wang B.G. (2017). Three new indole diterpenoids from the sea-anemone-derived fungus *Penicillium* sp. AS-79. Mar. Drugs.

[40] Yang B., He Y., Lin S., Zhang J., Li H., Wang J., Hu Z., Zhang Y. (2019). Antimicrobial dolabellanes and atranones from a marine-derived strain of the toxigenic fungus *Stachybotrys chartarum*. J. Nat. Prod..

[41] Miao F.P., Liang X.R., Yin X.L., Wang G., Ji N.Y. (2012). Absolute configurations of unique harziane diterpenes from *Trichoderma* species. Org. Lett..

[42] Liang X.R., Miao F.P., Song Y.P., Guo Z.Y., Ji N.Y. (2016). Trichocitrin, a new fusicoccane diterpene from the marine brown alga-endophytic fungus *Trichoderma citrinoviride* cf-27. Nat. Prod. Res..

[43] Li X.D., Li X., Li X.M., Xu G.M., Liu Y., Wang B.G. (2018). 20-nor-isopimarane epimers produced by *Aspergillus wentii* SD-310, a fungal strain obtained from deep sea sediment. Mar. Drugs.

[44] Xia X., Qi J., Liu Y., Jia A., Zhang Y., Liu C., Gao C., She Z. (2015). Bioactive isopimarane diterpenes from the fungus, *Epicoccum* sp. HS-1, associated with *Apostichopus japonicus*. Mar. Drugs.

[45] Kong F.D., Fan P., Zhou L.M., Ma Q.Y., Xie Q.Y., Zheng H.Z., Zheng Z.H., Zhang R.S., Yuan J.Z., Dai H.F. (2019). Penerpenes A-D, four indole terpenoids with potent protein tyrosine phosphatase iInhibitory activity from the marine-derived fungus *Penicillium* sp. KFD28. Org. Lett..

[46] Zhou L.M., Kong F.D., Fan P., Ma Q.Y., Xie Q.Y., Li J.H., Zheng H.Z., Zheng Z.H., Yuan J.Z., Dai H.F. (2019). Indole-diterpenoids with protein tyrosine phosphatase inhibitory activities from the marine-derived fungus *Penicillium* sp. KFD28. J. Nat. Prod..

[47] Li Y., Liu W., Han S., Zhang J., Xu W., Li Q., Cheng Z. (2020). Penitholabene, a rare 19-nor labdane-type diterpenoid from the deep-sea-derived fungus *Penicillium thomii* YPGA3. Fitoterapia.

[48] Fan Y., Wang Y., Liu P., Fu P., Zhu T., Wang W., Zhu W. (2013). Indole-diterpenoids with anti-H1N1 activity from the aciduric fungus *Penicillium camemberti* OUCMDZ-1492. J. Nat. Prod..

[49] Zhao D.L., Yang L.J., Shi T., Wang C.Y., Shao C.L., Wang C.Y. (2019). Potent phytotoxic harziane diterpenes from a soft coral-derived strain of the fungus *Trichoderma harzianum* XS-20090075. Sci. Rep..

[50] Ebada S.S., Schulz B., Wray V., Totzke F., Kubbutat M.H., Muller W.E., Hamacher A., Kassack M.U., Lin W., Proksch P. (2011). Arthrinins A-D: Novel diterpenoids and further constituents from the sponge derived fungus *Arthrinium* sp.. Bioorg. Med. Chem..

[51] Miao F.P., Liang X.R., Liu X.H., Ji N.Y. (2014). Aspewentins A-C, norditerpenes from a cryptic pathway in an algicolous strain of *Aspergillus wentii*. J. Nat. Prod..

[52] Song Y.P., Fang S.T., Miao F.P., Yin X.L., Ji N.Y. (2018). Diterpenes and sesquiterpenes from the marine algicolous fungus *Trichoderma harzianum* X-5. J. Nat. Prod..

[53] Niu S., Xie C.L., Xia J.M., Liu Q.M., Peng G., Liu G.M., Yang X.W. (2020). Botryotins A-H, tetracyclic diterpenoids representing three carbon skeletons from a deep-sea-derived *Botryotinia fuckeliana*. Org. Lett..

[54] Li H., Liu X., Li X., Hu Z., Wang L. (2021). Novel harziane diterpenes from deep-sea sediment fungus *Trichoderma* sp. SCSIOW21 and their potential anti-inflammatory effects. Mar. Drugs.

[55] Tsukada M., Fukai M., Miki K., Shiraishi T., Suzuki T., Nishio K., Sugita T., Ishino M., Kinoshita K., Takahashi K. (2011). Chemical constituents of a marine fungus, *Arthrinium sacchari*. J. Nat. Prod..

[56] Wang K.W., Ding P. (2018). New bioactive metabolites from the marine-derived fungi *Aspergillus*. Mini Rev. Med. Chem..

[57] Li X., Li X.-D., Li X.-M., Xu G.-M., Liu Y., Wang B.-G. (2017). Wentinoids A–F, six new isopimarane diterpenoids from *Aspergillus wentii* SD-310, a deep-sea sediment derived fungus. RSC Adv..

[58] Saha P., Rahman F.I., Hussain F., Rahman S.M.A., Rahman M.M. (2021). Antimicrobial Diterpenes: Recent Development from Natural Sources. Front. Pharmacol..

[59] Sun H.F., Li X.M., Meng L., Cui C.M., Gao S.S., Li C.S., Huang C.G., Wang B.G. (2012). Asperolides A-C, tetranorlabdane diterpenoids from the marine alga-derived endophytic fungus *Aspergillus wentii* EN-48. J. Nat. Prod..

[60] Qiao M.F., Ji N.Y., Liu X.H., Li K., Zhu Q.M., Xue Q.Z. (2010). Indoloditerpenes from an algicolous isolate of *Aspergillus oryzae*. Bioorg. Med. Chem. Lett..

[61] González M.C., Lull C., Moya P., Ayala I., Primo J., Primo Yúfera E. (2003). Insecticidal activity of penitrems, including penitrem G, a new member of the family isolated from *Penicillium crustosum*. J. Agric. Food. Chem..

[62] De Miccolis Angelini R.M., Rotolo C., Masiello M., Gerin D., Pollastro S., Faretra F. (2014). Occurrence of fungicide resistance in populations of *Botryotinia fuckeliana* (*Botrytis cinerea*) on table grape and strawberry in southern Italy. Pest Manag. Sci..

[63] Niu S., Peng G., Xia J.M., Xie C.L., Li Z., Yang X.W. (2019). A new pimarane diterpenoid from the *Botryotinia fuckeliana* fungus isolated from deep-sea water. Chem. Biodivers..

[64] Zhang M.Q., Xu K.X., Xue Y., Cao F., Yang L.J., Hou X.M., Wang C.Y., Shao C.L. (2019). Sordarin diterpene glycosides with an unusual 1,3-Dioxolan-4-one ring from the zoanthid-derived fungus *Curvularia hawaiiensis* TA26-15. J. Nat. Prod..

[65] Sun L., Li D., Tao M., Chen Y., Dan F., Zhang W. (2012). Scopararanes C-G: New oxygenated pimarane diterpenes from the marine sediment-derived fungus *Eutypella scoparia* FS26. Mar. Drugs.

[66] Mullowney M.W., Ó hAinmhire E., Tanouye U., Burdette J.E., Pham V.C., Murphy B.T. (2015). A pimarane diterpene and cytotoxic angucyclines from a marine-derived *Micromonospora* sp. in Vietnam’s east sea. Mar. Drugs.

[67] Gomes N.M., Bessa L.J., Buttachon S., Costa P.M., Buaruang J., Dethoup T., Silva A.M., Kijjoa A. (2014). Antibacterial and antibiofilm activities of tryptoquivalines and meroditerpenes isolated from the marine-derived fungi *Neosartorya paulistensis*, *N. laciniosa*, *N. tsunodae*, and the soil fungi *N. fischeri* and *N. siamensis*. Mar. Drugs.

[68] Gao S.S., Li X.M., Zhang Y., Li C.S., Wang B.G. (2011). Conidiogenones H and I, two new diterpenes of cyclopiane class from a marine-derived endophytic fungus *Penicillium chrysogenum* QEN-24S. Chem. Biodivers..

[69] Niu S., Fan Z., Tang X., Liu Q., Shao Z., Liu G., Yang X.-W. (2018). Cyclopiane-type diterpenes from the deep-sea-derived fungus *Penicillium commune* MCCC 3A00940. Tetrahedron Lett..

[70] Cheng Z., Li Y., Xu W., Liu W., Liu L., Zhu D., Kang Y., Luo Z., Li Q. (2019). Three new cyclopiane-type diterpenes from a deep-sea derived fungus *Penicillium* sp. YPGA11 and their effects against human esophageal carcinoma cells. Bioorg. Chem..

[71] Chen M.Y., Xie Q.Y., Kong F.D., Ma Q.Y., Zhou L.M., Yuan J.Z., Dai H.F., Wu Y.G., Zhao Y.X. (2021). Two new indole-diterpenoids from the marine-derived fungus *Penicillium* sp. KFD28. J. Asian Nat. Prod. Res..

[72] Zhao M., Ruan Q., Pan W., Tang Y., Zhao Z., Cui H. (2020). New polyketides and diterpenoid derivatives from the fungus *Penicillium sclerotiorum* GZU-XW03-2 and their anti-inflammatory activity. Fitoterapia.

[73] Li F., Sun W., Zhang S., Gao W., Lin S., Yang B., Chai C., Li H., Wang J., Hu Z. (2020). New cyclopiane diterpenes with anti-inflammatory activity from the sea sediment-derived fungus *Penicillium* sp. TJ403-2. Chin. Chem. Lett..

[74] Wang W., Wan X., Liu J., Wang J., Zhu H., Chen C., Zhang Y. (2018). Two new terpenoids from *Talaromyces purpurogenus*. Mar. Drugs.

[75] Su D., Ding L., He S. (2018). Marine-derived *Trichoderma* species as a promising source of bioactive secondary metabolites. Mini Rev. Med. Chem..

[76] Xie Z.-L., Li H.-J., Wang L.-Y., Liang W.-L., Liu W., Lan W.-J. (2013). Trichodermaerin, a new diterpenoid lactone from the marine fungus *Trichoderma erinaceum* associated with the sea star *Acanthaster planci*. Nat. Prod. Commun..

[77] Song Y.P., Liu X.H., Shi Z.Z., Miao F.P., Fang S.T., Ji N.Y. (2018). Bisabolane, cyclonerane, and harziane derivatives from the marine-alga-endophytic fungus *Trichoderma asperellum* cf44-2. Phytochemistry.

[78] Yamada T., Fujii A., Kikuchi T. (2019). New diterpenes with a fused 6-5-6-6 ring system isolated from the marine sponge-derived fungus *Trichoderma harzianum*. Mar. Drugs.

[79] Zou J.X., Song Y.P., Zeng Z.Q., Ji N.Y. (2021). Proharziane and harziane derivatives from the marine algicolous fungus *Trichoderma asperelloides* RR-dl-6-11. J. Nat. Prod..

[80] Ishino M., Kiyomichi N., Takatori K., Sugita T., Shiro M., Kinoshita K., Takahashi K., Koyama K. (2010). Phomactin I, 13-epi-phomactin I, and phomactin J, three novel diterpenes from a marine-derived fungus. Tetrahedron.

[81] Ishino M., Kinoshita K., Takahashi K., Sugita T., Shiro M., Hasegawa K., Koyama K. (2012). Phomactins K–M, three novel phomactin-type diterpenes from a marine-derived fungus. Tetrahedron.

[82] Ishino M., Kamauchi H., Takatori K., Kinoshita K., Sugita T., Koyama K. (2016). Three novel phomactin-type diterpenes from a marine-derived fungus. Tetrahedron Lett..

